# Effectiveness of Leg Elevation to Prevent Spinal Anesthesia-Induced Hypotension during Cesarean Delivery in the Resource-Limited Area: Open Randomized Controlled Trial

**DOI:** 10.1155/2020/5014916

**Published:** 2020-08-24

**Authors:** Sofia Assen, Bedru Jemal, Adane Tesfaye

**Affiliations:** ^1^Department of Anesthesia, College of Medicine and Health Sciences, Dilla University, Dilla, Ethiopia; ^2^Department of Public Health, College of Medicine and Health Sciences, Dilla University, Dilla, Ethiopia

## Abstract

**Background:**

Postspinal hypotension is the most common complication after spinal anesthesia for cesarean section (CS). Hypotension mainly occurs due to the reductions of vascular tone leading to decreased systemic vascular resistance and decreased venous return. The aim of this study was to assess the effectiveness of leg elevation (LE) as a method of prevention of postspinal hypotension in patients who undergo cesarean section under spinal anesthesia.

**Methods:**

This is a single-center parallel-randomized controlled trial study, and 52 full-term parturients scheduled for elective cesarean section who meets inclusion criteria were included in the study. The randomization sequence was created by a researcher not participating in patient management using a computer random generator. The participant was randomly assigned to the leg elevation group (*n* = 26) or to the control group (*n* = 26) of usual perioperative care.

**Results:**

The proportions of patients who develop hypotension are lower (8 (33.3%)) in the leg elevation group than the control group (15 (62.5%)) with an *X*^2^ (1, *N* = 48) = 4.09, *P*=0.043. The relative risk of developing postspinal hypotension in the leg elevation group compared to the control group was 0.47 (95% CI, 0.28–1.00). The proportion of severe hypotension was significantly decreased in the leg elevation group at a *P* value of 0.02.

**Conclusion:**

Performing leg elevation immediately after spinal anesthesia reduced the incidence of hypotension. The trial is registered with PACTR201908713181850.

## 1. Introduction

The global rate of cesarean section is estimated to be 15%. The prevalence of cesarean section is increasing from time to time in developing countries. According to a survey carried out in Ethiopia between December 2013 and January 2014, the prevalence of cesarean section is 19.2% in Addis Ababa, which is higher than the recommended rate in the WHO (10%–15%) [1].

Maternal hypotension is common following spinal anesthesia for cesarean section (CS), and its incidence reaches up to 60%–70%. Postspinal hypotension (PSH) in the cesarean section has been associated with adverse maternal and fetal outcomes. Severe hypotension poses a serious risk to mothers such as loss of consciousness, aspiration, and cardiac arrest and fetuses such as asphyxia and brain damage. PSH is mainly occurring due to the reductions of vascular tone leading to decreased systemic vascular resistance and decreased venous return [2–5].

Prevention and management of postspinal hypotension are continuously investigated. The incidence of hypotension during spinal anesthesia for cesarean section is reduced by administering intravenous fluids and vasopressors such as ephedrine, phenylephrine, norepinephrine, and mechanical technique such as leg compression and elevation mainly by increasing vascular tone and venous return. However, none of this single management protocol prevents the occurrence of hypotension [2, 6, 7].

Nowadays, the common clinical practice for prevention of spinal hypotension is by using a combination of different management protocols such as crystalloid coload and vasopressor administration before and during the procedure. Even though this is an effective method regarding to ensure maternal and fetal safety, it is not cost-effective and affordable in a resource-limited environment. Because of this, especially for developing countries, simple and cost-effective management protocol that can be easily applied with low experience and the less adverse effect is paramount in resource-limited areas [3, 4, 8].

Leg elevation (LE) creates an increase in venous return by translocation of blood from lower extremities to the thorax, which leads to increased stroke volume (SV) and consequently cardiac output (CO). LE is useful in settings with resource constraints such as our setup, and due to its simplicity and affordability, it avoids excessive expense [9, 10].

This study aims to assess the efficacy of leg elevation (LE) as a method of prevention of postspinal hypotension in patients who undergo elective cesarean section under spinal anesthesia.

## 2. Materials and Methods

### 2.1. Study Area

The study was conducted in Dilla University Referral Hospital, which is found in Dilla Town, Gedeo Zone, on the main road from Addis Ababa to Kenya, 360 km south of Addis Ababa, and 90 km south of Hawassa (capital of SNNPR). It is one of the public university hospitals providing health services to more than 4 million population of Gedeo Zone and neighboring catchment areas of Sidama and Oromia Region with 500 hospital beds. The hospital has four main departments (medical, surgical, pediatrics, and gynecology/obstetric wards), three special care units (medical intensive care unit, neonatal intensive care unit, and surgical recovery room), and five clinics (eye, antiretroviral treatment, dental, TB, and MDR-TB clinics).

### 2.2. Study Design and Period

The study was conducted from October 2018 to January 2019 at Dilla University Referral Hospital. The study design was a single-center parallel-randomized controlled trial recruiting 48 patients with equal proportions allocation.

#### 2.2.1. Source Population

The source population was all mothers who gave birth by elective cesarean section under spinal anesthesia in DURH.

#### 2.2.2. Study Population

The study population included mothers who gave birth by elective caesarian section under spinal anesthesia at DURH during the study period.

### 2.3. Inclusion Criteria

- Patients who are planned for the elective caesarian section under spinal anesthesia
- Age between 18 and 38 years

### 2.4. Exclusion Criteria

- Failed spinal block or total spinal converted to general anesthesia
- Those patients who have severe cardiac disease graded class (NYHA) III-IV
- Hypertensive disorders of pregnancy
- BMI ≥30 kg/m^2^
- Height <155 cm
- Preterm gestational age
- Multiple pregnancies

### 2.5. Sample Size

The sample size was calculated using G ^*∗*^ Power software version 3.1.9.2. Our primary outcome is the incidence of postspinal hypotension. Depending on the results of the previous study conducted by Hasanin et al., 2017, and M Obsaa et al, 2018, the reported incidence of postspinal hypotension in parturient with leg elevation and control was 34.7% and 80%, respectively. To detect a 45% absolute risk reduction in the incidence of postspinal hypotension, with a two-sided 5% significance level and power of 80%, a sample size of 26 per group was necessary, given an anticipated attrition rate of 10%.

### 2.6. Randomization

**Figure 1:**
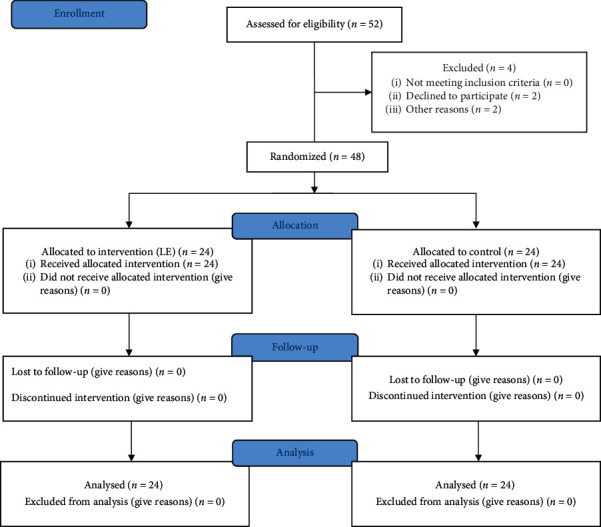
CONSORT diagram showing patient recruitment and flow.

The randomization sequence was created by a researcher not participating in patient management using a computer random generator. The participant was randomly assigned following the randomization procedure to one of two study groups. The allocation sequence was concealed from the researcher enrolling and assessing study participants (Figure 1).

### 2.7. Data Collection Procedures

Structured questionnaires were used to gather information from the patient's chart and mothers who underwent a cesarean section. Informed consent was taken, after descriptions of benefit, harm, and objectives of the study were informed to the patients. On arrival to the operating room, bilateral IV cannula was secured, and patients were premedicated with IV bolus of metoclopramide 10 mg and dexamethasone 4 mg before induction of anesthesia. After a brief settling period, baseline blood pressure and heart rate were recorded using an automated noninvasive (Anyveiw A8) monitoring device. All patients received 10 ml/kg normal saline coload over 15 minutes during spinal anesthesia as per our hospital protocol. Spinal anesthesia was initiated in the sitting position at the level of L3-L4 interspace by using Tuffier's line as a landmark. After the free flow of cerebrospinal fluid (CSF), 2.5 ml of 0.5%, isobaric bupivacaine (12.5 mg) was injected. The parturient was placed in supine and 15^0^ left lateral position, and supplemental oxygen was delivered by facemask at 4 L/min.

The intervention was given immediately after administration of spinal anesthesia, and for confirming the operating table was made straight, leg elevation was performed using two standard pillows positioned under the heel, so that the leg was elevated approximately 45° or 30 cm above the horizontal plane of the table. The controlled group was positioned in the regular supine position, and leg elevation stayed until end of surgery. To prevent cross-contamination, we covered the pillows by a plastic covering and changed for every patient.

Surgery was started just after confirmation of sensory and motor block. Sensory and motor blocks were tested by using a nontraumatic pinprick technique and the modified Bromage scale, respectively. Blood pressure was recorded at every 3-minute interval for the first 15 minutes after intrathecal injection and every five-minute interval until the end surgery. Postspinal hypotension was defined as systolic blood pressure (SBP) <80% of baseline reading. Hypotension was treated by increasing the rate of intravenous fluid administration and administering bolus phenylephrine incremental dose until SBP rise to 80% of the baseline value. Bradycardia was defined as 20% decrease in heart rate below baseline, and HR less than 60 bpm was managed by 0.5 mg intravenous atropine.

### 2.8. Statistical Analysis

Data were checked, coded, entered, and analyzed by using SPSS version 22 software packages. The data were tested for normality by using the Shapiro–Wilk normality test and homogeneity of variance by Levene's test for normally distributed. Numeric data will be described in terms of mean ± SD for symmetric and median (interquartile range) for asymmetric numeric data. Demographic data and preoperative variables were analyzed by using Student's *t*-test for normal distributed variables. Frequency and percentage were used to describe categorical variable, and statistical differences between groups were tested by using the chi-square test or Fisher exact test. A *P* value of less than 0.05 was considered as statistically significant.

### 2.9. Operational Definitions

  Hypotension: systolic blood pressure (SBP) <80% of baseline reading
  Bradycardia: a decrease in heart rate 20% from the baseline
  Postspinal hypotension: hypotension that occurs immediately after administration of local anesthetics
  Mild hypotension: SBP <80% of the baseline value
  Moderate hypotension: SBP <80–70% of the baseline value
  Severe hypotension: defined as SBP <70% of the baseline value
  Leg elevation (LE) group: the intervention group whose leg elevated around 30 cm (45 degrees), operating room table by using two standardized pillows placed under the heel of the patient immediately after taking spinal anesthesia
  Baseline: the average of the first three sets of measurements before induction anesthesia and starting operation
  Nausea and vomiting: when patients experience at least one episode of either nausea or vomiting within the period after giving SA until the end of surgery.

### 2.10. Ethical Consideration

Ethical clearance was obtained from the IRB of Dilla University College of Health Sciences and Medicine before the start of the study. The study protocol was approved by IRB. The data collector obtained informed written consent from each participant. Confidentiality was maintained at all levels of the study by avoiding identifiers and using codes to identify patients. Participant's involvement in the study was voluntary bases, and participants who were not willing to participate in the study and those who wish to quit their participation at any stage were informed to do so without any restriction.

This trial is registered with Pan African Clinical Trial Registry, under number PACTR201908713181850.

## 3. Result

### 3.1. Sociodemographic and Intraoperative Characteristics

Fifty-two patients were entered into the study from which there were four withdrawals. Two patients had inadequate block, which converted to general anesthesia, and two patients declined to participate in the study. The remaining twenty-four patients in each group were analyzed. The comparison of demographic and baseline vital signs shows no significant difference between groups (Table 1).

Intraoperative characteristics such as time from spinal anesthesia initiation to delivery of the fetus, duration of surgery, intraoperative fluid intake, estimated intraoperative blood loss, newborn weight, incidence of bradycardia, and nausea vomiting are comparable between the groups with a *P* value greater than 0.05 as given in Table 2.

### 3.2. Incidence of Postspinal Hypotension

**Figure 2:**
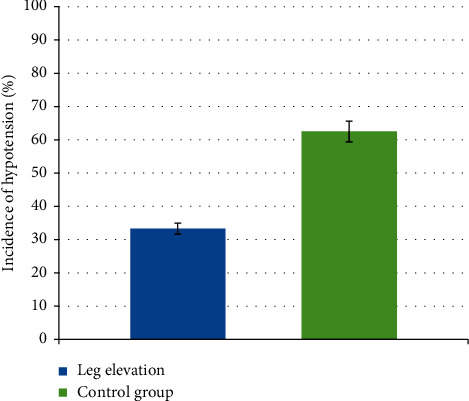
Incidence of postspinal hypotension between groups.

The incidence of postspinal hypotension in the leg elevation group shows a significant reduction. The proportions of patients who develop hypotension are lower 8 (33.3%) in the leg elevation group and compared to the control group 15 (62.5%) with an *X*^2^ (1, *N* = 48) = 4.09, *P*=0.02. The relative risk of developing postspinal hypotension in the leg elevation group compared to the control group was 0.47 (95% CI, 0.28–1.00). This means that leg elevation decreases the incidence of spinal-induced hypotension by 47% (Figure 2).

### 3.3. Severity of Postspinal Hypotension

**Figure 3:**
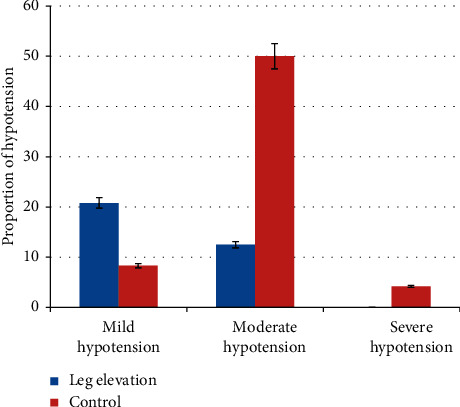
Severity of hypotension.

The incidence of severe postspinal hypotension in the leg elevation group was significantly lower compared to the control group *X*^2^ (1, *N* = 48) = 9.64, *P*=0.221 (Figure 3).

### 3.4. Intraoperative Total Phenylephrine Consumption

**Figure 4:**
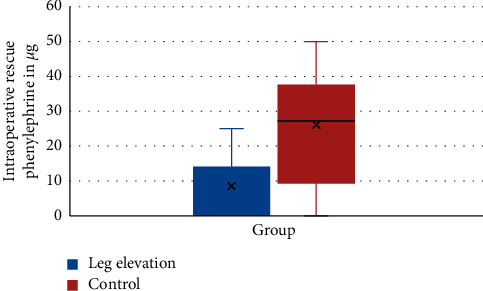
Intraoperative phenylephrine consumption.

Intraoperative rescue phenylephrine consumption between the leg elevation group and the control group did not differ significantly (25 ± 0 mg vs 36.4 ± 12.6, *P*=0.221) (Figure 4).

### 3.5. Intraoperative Hemodynamic Variable

**Figure 5:**
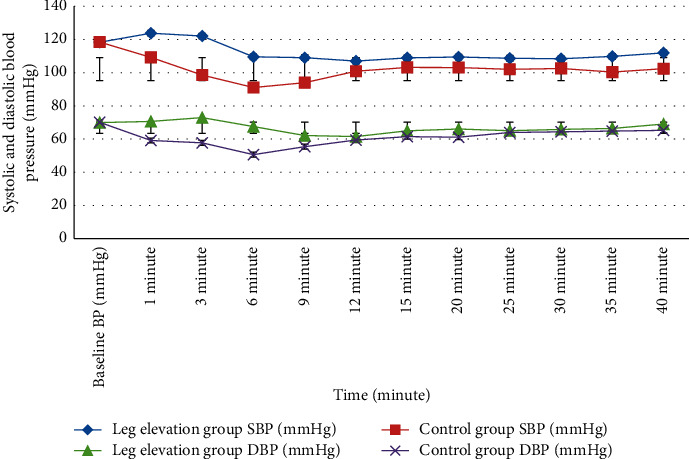
SBP and DBP between groups.

**Figure 6:**
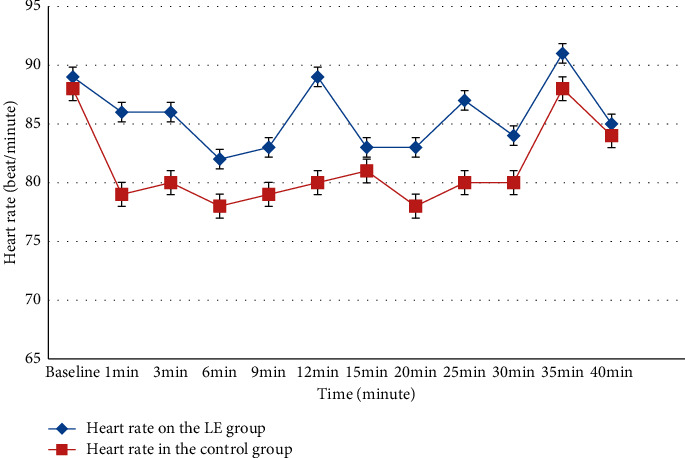
Heart rate between groups.

Changes in systolic and diastolic blood pressure over time are shown in Figure 5; intraoperative mean systolic and diastolic blood pressure trend in the leg elevation group maintained significantly higher blood pressure reading within the first ten minutes than in the control group (*P* < 0.05), whereas HR was similar between groups (*P* < 0.05) (Figure 6).

## 4. Discussion

Our study shows that the incidence of hypotension after spinal anesthesia was significantly lowered in the leg elevation group 33.3% compared to control 62.5%. The findings likely reflect the effect of augmentation in venous return due to leg elevation, which may lead to increased stroke volume and cardiac output. Additionally, leg elevation reduces risk of developing postspinal hypotension by 47%.

The result of our study is in line with the study performed in Egypt showing the lower incidence of postspinal hypotension in the leg elevation group compared to the control group. This randomized controlled trial demonstrates that the incidence of postspinal hypotension in the leg elevation group is 26 (34.7%) and 44 (58.7%) in the control group with a *P* value of 0.05. The relative risk reduction of leg elevation was 40.9% with 95% CI, 0.193–0.724 [8, 11]. The result of our study was in line with this study. Relative risk reduction of our study is by 47%, which is statistically significant.

In contrary to our study, a randomized controlled trial performed in England by Rout et al. did not show the effect of leg elevation on the reduction of postspinal hypotension. The study was performed on 31 patients in each group. The incidence of PSH in leg wrapped, leg elevation, and controlled group, respectively, was 18%, 39%, and 53% with a *P* value of 0.004. Rout et al.' study confirmed a significant reduction in the incidence of postspinal hypotension by leg wrapping compared to leg elevation alone. Leg elevation alone did not show a statistically significant reduction of the incidence of postspinal hypotension with 95% CI, 0.7–4.9 [12]. The possible explanation for this contradictory result is a difference in population and hypotension management practice in the study set up.

Our study demonstrates that median phenylephrine consumption was lower in the leg elevation group compared to the control group, but significant statistical difference was not observed between groups. Our finding is comparable with the study performed in England, which shows that the mean consumption of ephedrine were 18.5 ± 13.6, 10.8 ± 5.15, and 16.7 1 ± 2.5 in the control group, leg elevation group, and leg wrapped group, respectively, which was not significant [12]. In contrary to our study, the study conducted by Hasnan et al. shows that leg elevation reduces total vasopressor consumption, and the total ephedrine consumption was 4.9 ± 7.8 mg in the leg elevation group and 10 ± 11 mg in the controlled group, which is statistically significant [11]. The reason for the difference in result may be due to pharmacologic difference used for management of hypotension after spinal anesthesia, and larger sample may be attributed to the difference.

Our finding shows the overall incidence of nausea and vomiting after spinal anesthesia was higher in the control group compared to the leg elevation group, but the difference was not statistically significant. This shows a similar result compared to a study performed by Hasnan et al. where the incidence of nausea and vomiting was not significantly different between groups.

Finally, limitations of this study were on hemodynamic assessment, which only depends on heart rate and blood pressure. We believe that the use of cardiac output monitors and invasive monitoring in future studies might be more informative to identify the precise effect of leg elevation on maternal hemodynamics.

In summary, this study shows that performing leg elevation after spinal anesthesia for elective cesarean section significantly reduces the incidence of postspinal hypotension and severity of hypotension.

## Figures and Tables

**Table 1:** Demographic and operative characteristics.

	Leg elevation group(*n* = 24)	Control group	*P* value
Age (years)	27.6 ± 3	26.9 ± 4	0.455
Height (cm)	1.66 ± 2	1.64 ± 7	0.55
Weight (kg)	60 ± 2	62 ± 2	0.57
BMI (kg/m^2^)	24 ± 1	23.5 ± 1	0.597
Baseline systolic BP (mmHg)	118 ± 9	119 ± 9.6	0.963
Baseline diastolic BP (mmHg)	70 ± 6	71 ± 7	0.932
Baseline HR	89 ± 6	88.7 ± 5	0.756
Number of previous C/S			0.67
0	13 (54.2%)	10 (41.7%)	
1	5 (20.8%)	7 (29.2%)	
2	6 (25%)	7 (29.2%)	

**Table 2:** Intraoperative characteristics of patients who underwent elective cesarean.

	Leg elevation group (*n* = 24)	Control group (*n* = 24)	*P* value
Time from SAB to delivery of the fetus in minutes	17 ± 1.8	16.5 ± 1.4	0.172
Duration of surgery in (min)	38.7 ± 3.6	39 ± 3.4	0.809
Intraoperative fluid (ml)	1841.6 ± 150.8	1939.5 ± 289	0.096
Weight of baby	3.5 ± 0.7	3.4 ± 0.4	0.543
Incidence of nausea and vomiting	2 (8.3%)	5 (20%)	0.416
Incidence of bradycardia	1 (4.2%)	2 (8.3%)	0.551
Blood loss	582 ± 91.8	551 ± 81	0.149
Number of patients who take phenylephrine	3 (12.5%)	13 (54.5%)	0.02
Total atropine consumption (mg)	0.5 ± 0	0.7 ± 0.3	0.667

## Data Availability

The data used to support the findings of this study are available on request.

## Abbreviations

BP: Blood pressure
CI: Confidence interval
CS: Cesarean section
CSF: Cerebrospinal fluid
DURH: Dilla University Referral Hospital
HR: Heart rate
IV: Intravenous
L: Lumbar vertebrae
LE: Leg elevation
MAP: Mean arterial pressure
NIBP: Noninvasive blood pressure
NYHA: New York Heart Association
PSH: Postspinal hypotension
RCT: Randomized control trial
SA: Spinal anesthesia
T: Thoracic vertebrae.

## References

[1] Bayou Y. T., Mashalla Y. J. S., Thupayagale-Tshweneagae G. (2016). Patterns of caesarean-section delivery in Addis Ababa, Ethiopia. *African Journal of Primary Health Care & Family Medicine*.

[2] Cyna A. M., Andrew M., Emmett R. S., Middleton P., Simmons S. W. (2006). Techniques for preventing hypotension during spinal anaesthesia for caesarean section. *Cochrane Database of Systematic Reviews*.

[3] Loubert C. (2012). Fluid and vasopressor management for Cesarean delivery under spinal anesthesia: continuing professional development. *Canadian Journal of Anesthesia/Journal Canadien D’anesthésie*.

[4] Mercier F. J., Augé M., Hoffmann C., Fischer C., Le Gouez A. (2013). Maternal hypotension during spinal anesthesia for cesarean delivery. *Minerva Anestesiol*.

[5] Shahzadi I., Hanif S., Afridi Y. (2016). Role of ephedrine infusion in spinal anaesthesia induced hypotension. *Pakistan Journal of Physiology*.

[6] Cluver C., Novikova N., Hofmeyr G. J., Hall D. R. (2013). Maternal position during caesarean section for preventing maternal and neonatal complications. *Cochrane Database of Systematic Reviews*.

[7] Ngan Kee W. D., Khaw K. S. (2006). Vasopressors in obstetrics: what should we be using?. *Current Opinion in Anaesthesiology*.

[8] Hasanin A., Mokhtar A. M., Badawy A. A., Fouad R. (2017). Post-spinal anesthesia hypotension during cesarean delivery, a review article. *Egyptian Journal of Anaesthesia*.

[9] Caille V., Jabot J., Belliard G., Charron C., Jardin F., Vieillard-Baron A. (2008). Hemodynamic effects of passive leg raising: an echocardiographic study in patients with shock. *Intensive Care Medicine*.

[10] Wong D. H., Tremper K. K., Zaccari J., Hajduczek J., Konchigeri H. N., Hufstedler S. M. (1988). Acute cardiovascular response to passive leg raising. *Critical Care Medicine*.

[11] Hasanin A., Aiyad A., Elsakka A. (2017). Leg elevation decreases the incidence of post-spinal hypotension in cesarean section: a randomized controlled trial. *BMC Anesthesiology*.

[12] Rout C. C., Rocke D. A., Gouws E. (1993). Leg elevation and wrapping in the prevention of hypotension following spinal anaesthesia for elective caesarean section. *Anaesthesia*.

